# Supplementary material for: A Yellow Fever Virus 17D Infection and Disease Mouse Model Used to Evaluate a Chimeric Binjari-Yellow Fever Virus Vaccine
Source: Vaccines (Basel). 2020 Jul 9;8(3):368. doi: 10.3390/vaccines8030368 (PMC7564786; doi:10.3390/vaccines8030368)
Supplement: Supplementary file 1 [file vaccines-08-00368-s001.pdf]

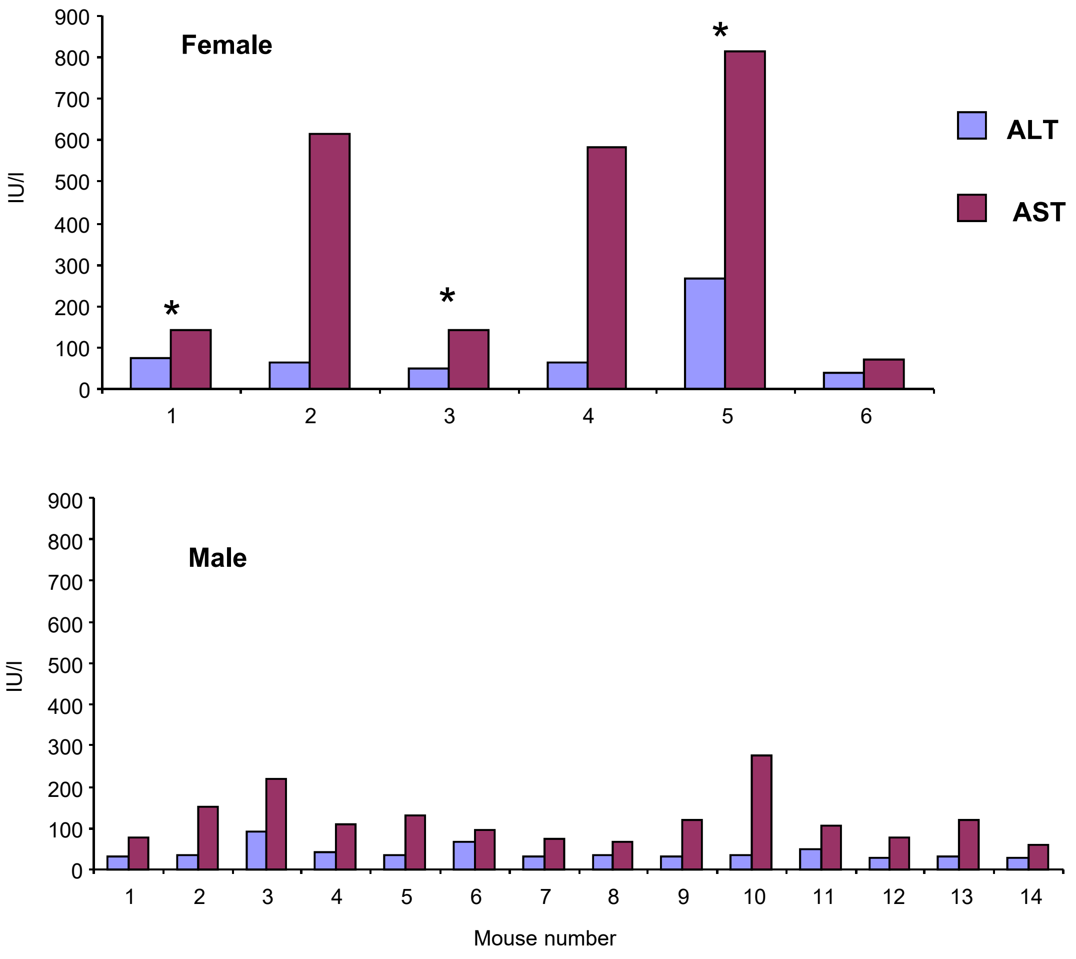

Figure S1. AST and ALT levels in blood day 6 post YFV 17D infection undertaken by IDEXX Laboratories, Brisbane, Australia. ALT levels >60 and AST levels >100 might be considered clinically significant, with control mice routinely showing levels below these values. Overall mean levels were not significantly different between male and female mice.

* indicates mice with overt white necrotic liver lesions

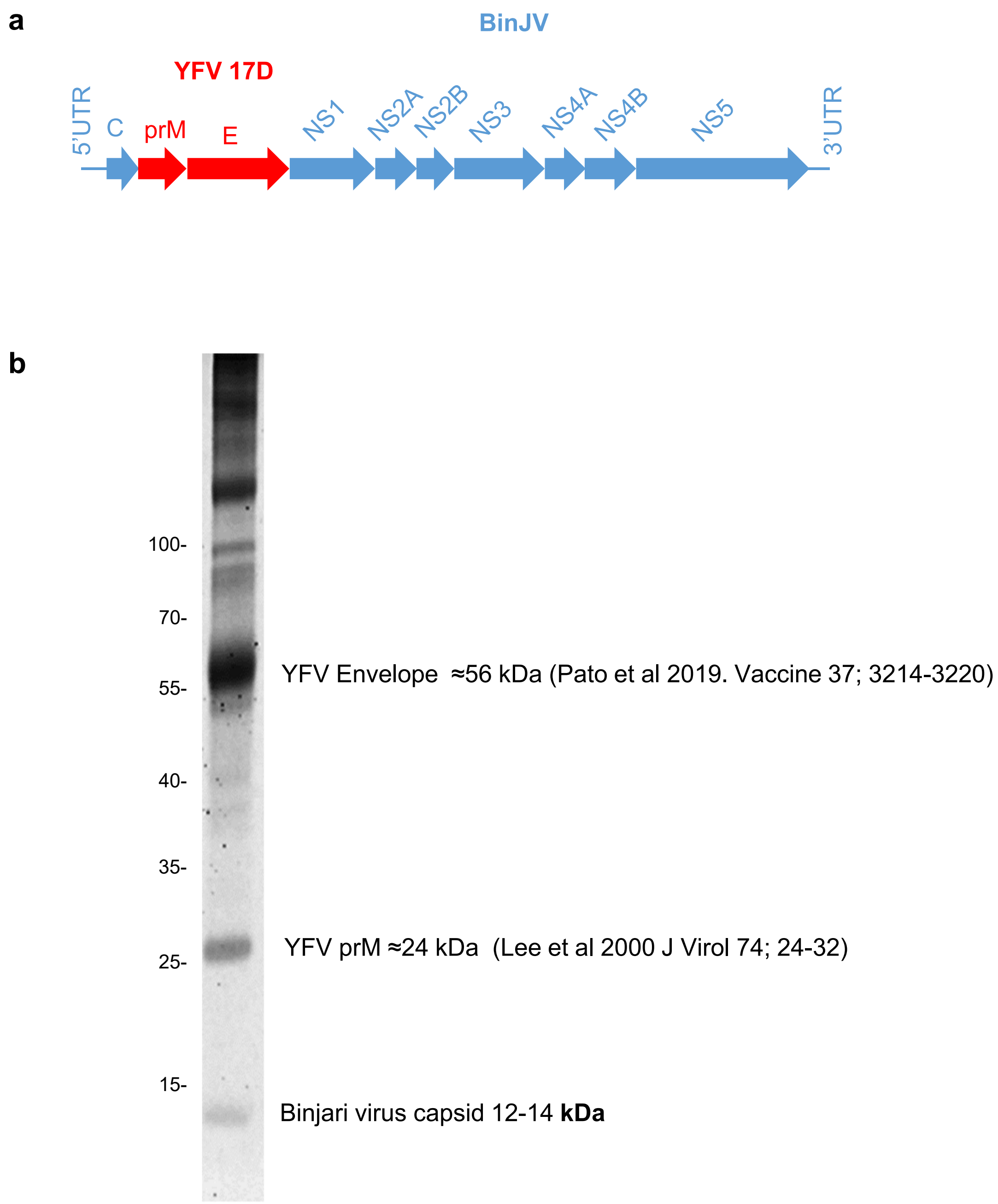

Figure S2. **a** The chimeric BinJ/YFV-prME construct. **b** SDS PAGE of the BinJ/YFV-prME vaccine preparation indicating major structural protein bands .

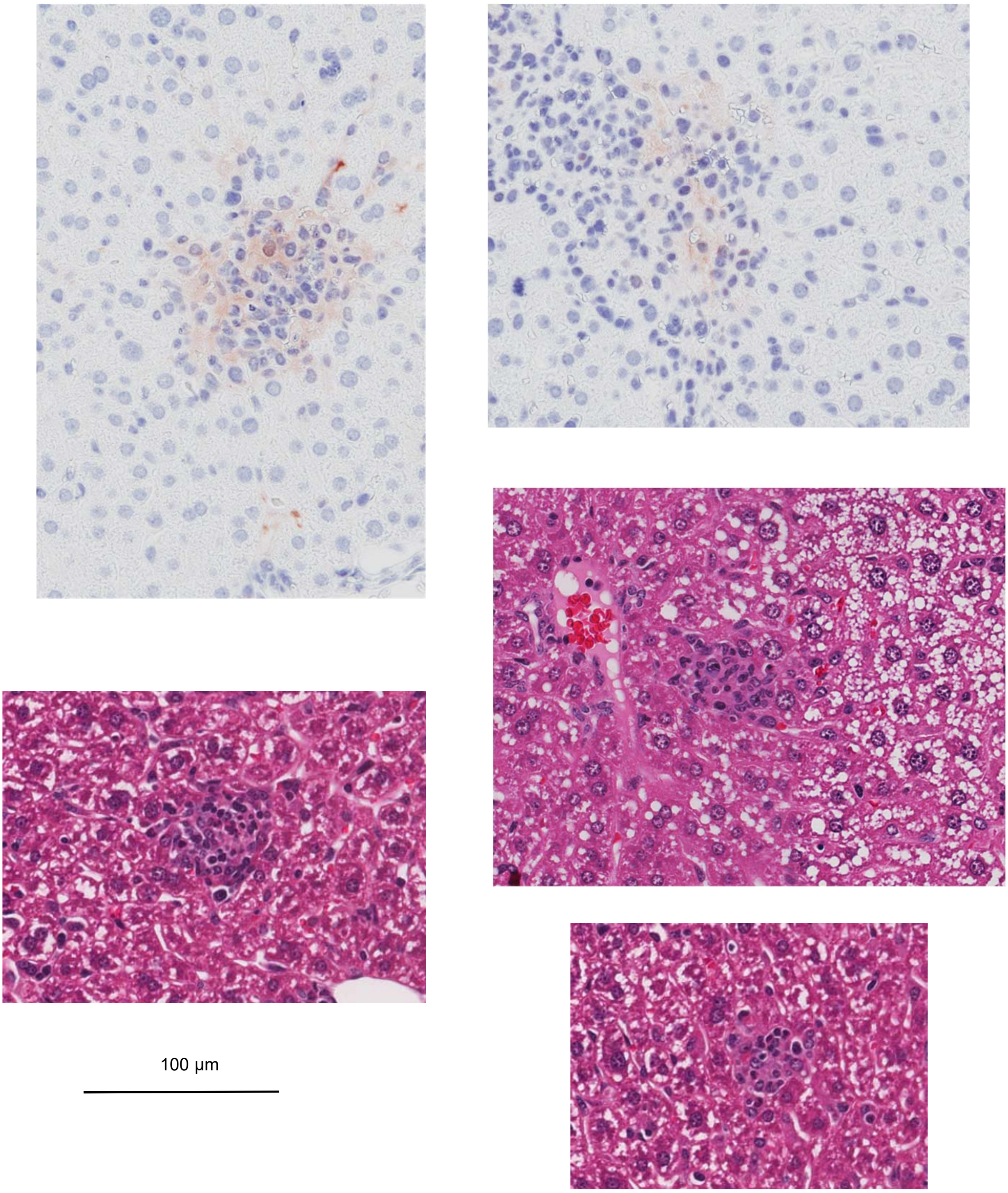

Figure S3. Examples of infiltrate foci by IHC and H&E staining of liver sections from control mice infected with YFV 17D

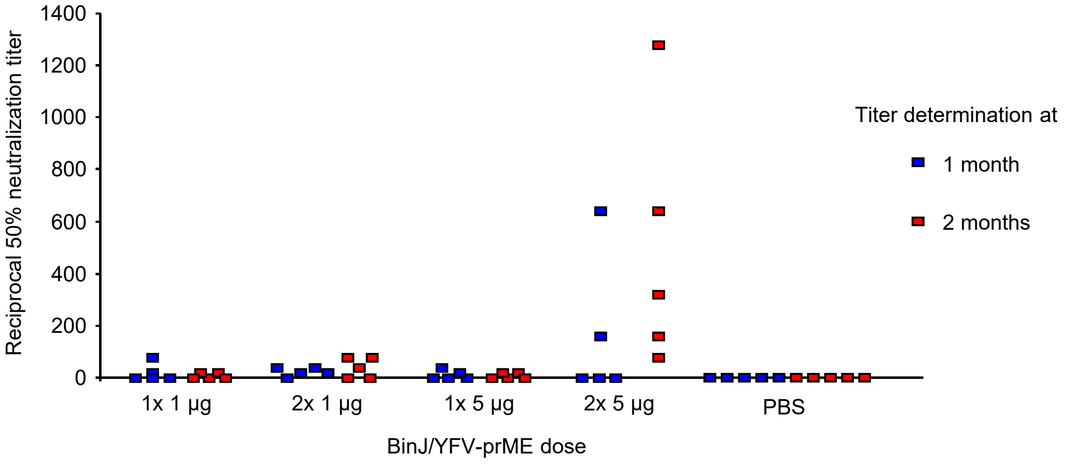

Figure S4. CD1 mice (male and female), 5 weeks old at first vaccination. Booster (2x) given 3 weeks after first vaccination. Neutralization titers determined at 1 month (Blue) and 2 months (Red) after the last vaccination.
